# OLFACTORY DYSFUNCTION AND DEPRESSION TRAJECTORIES IN COMMUNITY-DWELLING OLDER ADULTS

**DOI:** 10.1093/geroni/igad104.0629

**Published:** 2023-12-21

**Authors:** Vidyulata Kamath, Kening Jiang, Kevin Manning, R Mackin, Keenan Walker, Willa Brenowitz, Eleanor Simonsick, Jennifer Deal

**Affiliations:** Johns Hopkins University School of Medicine, Baltimore, Maryland, United States; Johns Hopkins Bloomberg School of Public Health, Baltimore, Maryland, United States; University of Connecticut Health, Farmington, Connecticut, United States; University of California, San Francisco, California, United States; National Institute on Aging, National Institutes of Health, Baltimore, Maryland, United States; University of California, San Francisco, California, United States; National Institute on Aging, National Institutes of Health, Baltimore, Maryland, United States; Johns Hopkins Bloomberg School of Public Health, Baltimore, Maryland, United States

## Abstract

Olfactory dysfunction has been linked with all-cause mortality and neurodegenerative disease risk, though less is known about the association between olfaction and late-life depression (LLD). We examined the relationship between baseline olfactory performance and incident significant depressive symptoms and longitudinal depression trajectories in well-functioning older adults from the Health, Aging, and Body Composition study. Older adults (n=2,125, age 71-82, 51% women, 37% Black) completed an odor identification task at Year 3 (our study baseline). Cognitive assessments and depressive symptoms were ascertained across multiple visits over 8 years. We employed discrete time complementary log-log models, group-based trajectory models and multivariable-adjusted multinomial logistic regression to assess the relationship between baseline olfaction and incident depression and longitudinal depression trajectories. Mediation analysis assessed the influence of cognition on these relationships. Individuals with lower olfaction had increased risk of developing significant depressive symptoms at follow-up (hazard ratio [HR]=1.04, 95% CI:1.00, 1.08). Of the three longitudinal depression score patterns identified (stable low, stable moderate, stable high), poorer olfaction was associated with a 6% higher risk of membership in the stable moderate (relative risk ratio [RRR]=1.06, 95% CI:1.02, 1.10) and stable high (RRR=1.06, 95% CI:1.00, 1.12) groups, compared to the stable low group. Poor cognitive status only partially mediated the relationship between olfaction and incident depression severity. Suboptimal olfaction may serve as a prognostic indicator of vulnerability for development of LLD. These findings underscore the need for a greater understanding of olfaction in LLD and the demographic, cognitive and biological factors that influence these relationships over time.

